# A comparison of preliminary oncologic outcome and postoperative complications between patients undergoing either open or robotic radical cystectomy

**DOI:** 10.1590/S1677-5538.IBJU.2015.0393

**Published:** 2016

**Authors:** Antonio Cusano, Peter Haddock, Max Jackson, Ilene Staff, Joseph Wagner, Anoop Meraney

**Affiliations:** 1 Urology Division, Hartford Healthcare Medical Group, Hartford, USA

**Keywords:** Oncology Nursing, Postoperative Period, Robotics, Cystectomy, Urinary Bladder Neoplasms

## Abstract

**Purpose:**

To compare complications and outcomes in patients undergoing either open radical cystectomy (ORC) or robotic-assisted radical cystectomy (RRC).

**Materials and Methods:**

We retrospectively identified patients that underwent ORC or RRC between 2003- 2013. We statistically compared preliminary oncologic outcomes of patients for each surgical modality.

**Results:**

92 (43.2%) and 121 (56.8%) patients underwent ORC and RRC, respectively. While operative time was shorter for ORC patients (403 vs. 508 min; p<0.001), surgical blood loss and transfusion rates were significantly lower in RRC patients (p<0.001 and 0.006). Length of stay was not different between groups (p=0.221). There was no difference in the proportion of lymph node-positive patients between groups. However, RRC patients had a greater number of lymph nodes removed during surgery (18 vs. 11.5; p<0.001). There was no significant difference in the incidence of pre-existing comorbidities or in the Clavien distribution of complications between groups.

ORC and RRC patients were followed for a median of 1.38 (0.55-2.7) and 1.40 (0.582.59) years, respectively (p=0.850). During this period, a lower proportion (22.3%) of RRC patients experienced disease recurrence vs. ORC patients (34.8%). However, there was no significant difference in time to recurrence between groups. While ORC was associated with a higher all-cause mortality rate (p=0.049), there was no significant difference in disease-free survival time between groups.

**Conclusions:**

ORC and RRC patients experience postoperative complications of similar rates and severity. However, RRC may offer indirect benefits via reduced surgical blood loss and need for transfusion.

## INTRODUCTION

Bladder cancer is the fourth most commonly diagnosed malignancy in the United States, with a peak incidence between 50-70 years of age (1). Open radical cystectomy (ORC) has been considered the gold-standard of care for the surgical treatment of bladder cancer (2). However, following the first reported robotic-assisted laparoscopic cystectomy (RRC) more than a decade ago (2), its positive effects on reducing surgical blood loss, accelerating recovery time, and shortening surgical learning times have been reported.

A prospective, randomized trial of 124 patients failed to identify significant advantages of RRC compared to ORC in terms of 90 day complication rates, length of stay, pathologic outcome and short-term quality of life measures (3). However, neither the rate of recurrence or time to all-cause death were compared between surgical modalities.

While these clinical data have supported the wider use of RRC (4, 5), comprehensive studies comparing clinical outcomes between patients undergoing ORC or RRC have only recently started to emerge. In addition, the majority of published reports and randomized clinical trials comparing open and robotic surgical modalities have been published from NCI comprehensive programs or from community cancer network sites, rather from patients treated at a large community hospital.

In the present study, we retrospectively compared oncologic outcome, post-operative complication rates, disease recurrence and all-cause mortality in patients who underwent either open or robotic-assisted radical cystectomy during a 10-year contemporary period.

## MATERIALS AND METHODS

A retrospective review of our IRB-approved cystectomy database identified 213 patients with demographic and clinical data who underwent radical cystectomy between January 2003-December 2013. These patients were stratified on the basis of whether they underwent ORC or RRC. Four surgeons performed the open surgeries, while two surgeons performed the robotic procedures. The choice of surgery type was based upon consultation and between the patient and physician, comorbid conditions, tumor/pathologic stage, and also the availability of robotic surgical suites. In the early phase of our study period (2003-2008), >80% of cases were performed using an open procedure. In more recent years (2009-2013), however, the majority of surgeries (66%) were robotic-assisted, which is likely a reflection of surgeons becoming more confident with robotic procedures. Although in most robotic cases urinary diversion was created extracorporeally, intracorporeal urinary diversions also was created in few, more recent, cases.

Patient demographics and clinical data including preexisting comorbidities, Charlson Comorbidity Index (CCI) score, chemotherapy history and peri/postoperative complications that occurred ≤90 days post-surgery were tabulated. Follow-up intervals were every 3 months for the first 2 years, then every 6 months for the next 3 years, and annually thereafter. Complication severity was classified according to the Clavien grading system (6). Clinical tumor stage was defined from MRI/CT imaging and cystoscopy/TURBT pathology prior to surgery. The association between risk factors, comorbidities, CCI score, and patient outcomes for each surgical type were evaluated. Recurrence was primarily determined at follow-up through work up, imaging (e.g. CT scan), cytology and/or biopsy.

The incidence of complications following ORC and RRC was compared. Continuous variables were compared utilizing independent samples t-test or Wilcoxon Ranked Sum tests depending on whether assumptions concerning the underlying distributions were met. Chi-square tests of proportion were utilized to compare data for dichotomous and categorical variables. Kaplan-Meier analyses were used to determine time to disease recurrence and disease-free survival time. All statistical analyses were performed using SPSS v21.0 (SPSS, Inc., Chicago, IL, USA).

## RESULTS

### All cystectomy patients

A total of 213 patients who underwent either ORC or RRC procedures between January 2003-December 2013 formed the study cohort, of which 168 (78.9%) were male (Table-1). The mean age of the entire study cohort was 66.7±10.4 years. A total of 141 (66.2%) of patients were either current or former tobacco users. In addition, 39 (18.3%) of patients had pre-operative hydronephrosis. Neoadjuvant therapy was used in 59 (27.7%) patients, while adjuvant chemotherapy was utilized in 55 (25.8%) patients across both surgery types. A total of 62 (29.1%) tumors were clinically staged ≤cT1, while 144 (67.6%) were staged cT2-cT4. A total of 27 (12.7 %) patients were downstaged (≥T2 to ≤Ta/Tis) on final pathology.

**Table 1:** Demographic, surgical and oncologic data.

			Cystectomy procedure		
			Open	Robotic	P
**Demographic data**					
	Number of patients (n; %)		92 (43.2)	121 (56.8)	-
	Age at surgery (mean ± SD) (years)		67.8±10.4	65.9±10.4	0.176
	Gender (male; %)		73 (79.3)	95 (78.5)	1.0
	Tobacco use (ever smoked) (n; %)		70 (89.7)	71 (65.1)	<0.001
	BMI(mean ± SD)(kg/m^2^)		28.4±5.2	28.2±5.0	0.860
	CCI score		4 (3-5)	4 (3-5)	0.694
	ASA physical status score		3 (2-3)	3(2-3)	0.407
	Patients with pre-operative hydronephrosis (n; %)		18(19.6)	21 (17.4)	0.722
	Patients who received adjuvant chemotherapy (n; %)		21 (22.8)	38(31.4)	0.402
	Follow up time (years)		1.38(0.55-2.7)	1.40(0.58-2.59)	0.850
**Surgical and oncologic data**					
	Estimated blood loss (median; IQR) (mL)		600(450-1100)	450 (300-725)	<0.001
	Patients requiring transfusion (n; %)		36(39.1)	26(21.5)	0.006
	Operative time (median; IQR) (min)		403 (359-467)	508 (436-589)	<0.001
	Length of stay (median; IQR) (days)		9(7-13.8)	8(7-12)	0.221
	Patients with disease recurrence (n; %)		32 (34.8)	27 (22.3)	0.046
	Patients with positive margins (n; %)		5 (5.6)	10(8.3)	0.591
	Lymph node-positive patients (n; %)		22 (24.4)	25 (20.7)	0.514
	Median number of lymph nodes removed from all patients		11.5(7-19)	18(11-24)	<0.001
	Positive lymph nodes removed from lymph patients node-positive patients		2(1-3)	2.5(1-4.2)	0.354
	Total patients down-staged (≥T2 to ≤Ta/Tis (n; %) on final pathology) (n; %)		7 (7.6)	20(16.5)	0.062
	Patients down-staged who received adjuvant chemotherapy (n; %)		4(19.0)	11 (28.9)	0.045
	Number of deaths (all causes) (n; %)		34 (37.0)	29 (24.0)	0.049
Lymphovascular invasion (n; %)		TURB specimen	17(18.9)	23(19.0)	1.0
		Cystectomy specimen	30 (32.6)	38(31.4)	0.883
		Ileal conduit	64 (69.6)	71 (59.2)	
Type of diversion (n; %)		Indiana pouch	5 (5.4)	13(10.8)	0.208
		Neobladder	23 (25.0)	36 (30.0)	

Data are shown as median (interquartile range) unless otherwise specified.

### Comparison of open and robotic radical cystectomy patients

Demographics: When stratified by surgical modality, a total of 92 (43.2%) and 121 (56.8%) patients underwent ORC and RRC, respectively. There was no significant difference in the distribution of clinical or pathologic tumor stages between patients who underwent ORC and RRC (p=0.437 and 0.267; Tables 2 and 3). In addition, there was no significant difference in the distribution of the type of urinary diversion (i.e. ileal conduit, Indiana pouch and neobladder) utilized in ORC and RRC patients (p=0.208; Table-1).

**Table 2:** Outcome stratified by clinical stage.

		Cystectomy procedure		
		Open	Robotic	P
	≤cT1	25 (27.5)	37 (30.8)	
Clinical bladder cancer stage (n; %)	cT2	45 (49.5)	65 (54.2)	0.437
	cT3	15(16.5)	11 (9.2)
	cT4/node positive (N1/N2)	6 (6.6)	7 (5.8)	
Patients with muscle-invasive tumors (n: %)		66 (72.5)	83 (69.2)	0.649
	All stages	12.6(9.3-15.9)	11.8(7.7-15.9)	0.949
	≤cT1	17.3(9.6-25.1)	13.2(4.5-21.8)	0.858
Time to recurrence (mean; 95% CI) (months)	cT2	15.9(10.8-20.9)	12.8(7.8-17.8)	0.318
	cT3	5.8 (2.8-8.7)	16.0(0.0-39.6)	0.410
	cT4/node positive (N1/N2)	5.0 (2.7-7.3)	3.3(0.01-6.5)	0.841
	All stages	66.9 (55.3-78.6)	68.8 (59.6-77.9)	0.301
	≤cT1	74.3 (54.7-93.9)	67.5(57.1-77.9)	0.531
Time to all-cause death (mean; 95% CI) (months)	cT2	74.4 (58.8-90.0)	72.4 (59.9-84.9)	0.416
	cT3	24.6(14.2-35.1)	26.6(18.5-34.8)	0.459
	cT4/node positive (N1/N2)	50.2(16.6-83.7)	30.3(14.4-46.2)	0.864

**Table 3:** Outcome stratified by pathologic stage.

		Cystectomy procedure		
		Open	Robotic	P
Pathologic bladder cancer stage (n; %)	≤pT1	29(31.5)	50(41.3)	0.267
	pT2	25 (27.2)	22(18.2)	
	pT3	25 (27.2)	28(23.1)	
	pT4/node positive (N1/N2)	13(14.1)	21 (17.4)	

The age of ORC and RRC patients at the time of surgery was not significantly different (67.8±10.4 versus 65.9±10.4 years, respectively; p=0.176, Table-1). ORC and RRC patients were followed for a median of 1.38 (0.55-2.7) and 1.40 (0.58-2.59) years, respectively (p=0.850). There was also no significant difference in gender distribution, BMI, CCI score and ASA score between ORC and RRC patient cohorts (p=1.0, 0.860, 0.694, 0.407, respectively; Table-1). A total of 70 (89.7%) ORC patients had a history of tobacco use, which was significantly higher than the proportion of RRC patients who were tobacco users (p<0.001; Table-1). There was no significant difference in the utilization of either neoadjuvant or adjuvant chemotherapy between ORC and RRC patients (p=0.166 and 0.402, respectively). In addition, the proportion of patients with pre-operative hydronephrosis was not statistically different between patients undergoing ORC and RRC (p=0.722; Table-1).

Surgical and oncologic outcome: There was no significant difference in the distribution of the type of urinary diversion performed in ORC and RRC patients (p=0.208; Table-1).

There was also no significant difference in the incidence of muscle-invasive tumors between surgical groups, based on either their clinical or pathologic staging (p=0.649 and 0.154, respectively; Tables 2 and 3). Of the patients who received neoadjuvant chemotherapy, 4/21 (19.0%) of ORC and 11 (28.9%) of RRC patients were downstaged on final pathology (p=0.045).

There was no significant difference in the proportion of ORC and RRC patients with lympho-vascular invasion (based on either TURB or cystectomy specimens) (Table-1). There was also no significant difference in the number of positive margins on biopsy between surgical groups (p=0.591; Table-2), or in the proportion of lymph node-positive patients (p=0.514; Table-1). However, patients who underwent RRC had a significantly greater number of lymph nodes removed than those undergoing ORC (18 versus 11.5; p<0.001; Table-1). There was no significant difference in the number of positive lymph nodes removed from lymph-node-positive patients between ORC and RRC groups (2 versus 2.5; p=0.354).

Recurrence and mortality: A significantly lower proportion of patients who underwent RRC experienced disease recurrence than patients who underwent open surgery (22.3 versus 34.8%; p=0.046; Table-1). However, there was no significant difference in the time to recurrence between ORC and RRC patients, based on either clinical or pathologic stage (Tables 2 and 3). A significantly higher all-cause death rate was observed in patients undergoing ORC compared to RRC (37 versus 24%; p=0.049; Table-1). However, there was no significant difference in the time to all-cause death between ORC and RRC patients, when stratified by either clinical or pathologic tumor stage (Tables 2 and 3).

Comorbidities and peri/postoperative complications: Estimated blood loss during RRC was significantly lower than in patients undergoing ORC (450 versus 600mL; p<0.001; Table-1). Furthermore, a significantly lower proportion of RRC patients required transfusion compared to those undergoing ORC (21.5 versus 39.1%; p=0.006; Table-1). Estimated surgical blood loss in patients requiring a transfusion was not significantly different between open and robotic surgeries (p=0.635). However, operative time for ORC was significantly shorter than for RRC (403 versus 508 min; p<0.001; Table-1). Length of hospital stay was not significantly different between the two surgical groups (p=0.221; Table-1). There was no significant difference in the incidence of pre-existing comorbidities between ORC and RRC patient groups (Table-4).

**Table 4:** Incidence of comorbidities and complications.

		Cystectomy procedure		
		Open	Robotic	P
	Coronary artery disease	15(16.3)	17(14)	0.701
	Chronic heart failure	2 (2.2)	2(1.7)	1.0
	Chronic obstructive pulmonary disorder	10(10.9)	19(15.7)	0.420
	Diabetes	17(18.5)	23(19.0)	1.0
	Connective tissue	10(10.9)	12(9.9)	0.824
	Peptic ulcer	2 (2.2)	2(1.7)	1.0
Comorbidities (n; %)	Renal insufficiency	7 (7.6)	8 (6.6)	0.793
	Cerebrovascular accident	3 (3.3)	6 (5.0)	0.735
	Peripheral vascular disease	4 (4.3)	4 (3.3)	0.729
	Lymphoma	0(0)	2(1.7)	0.507
	Myocardial infarction	9 (9.8)	4 (3.3)	0.080
	Liver	1(1.1)	0(0)	0.432
	Any	44 (47.8)	63(52.1)	0.581
	Cardiac	10(10.9)	5(4.1)	0.064
	Respiratory	5 (5.4)	7 (5.8)	1.0
	Genitourinary	6 (6.5)	6 (5.0)	0.766
	Gastrointestinal	23 (25.0)	24(19.8)	0.406
	Infection	13(14.1)	16(13.2)	0.843
	Vascular	15(16.3)	16(13.2)	0.560
Complications (n; %)	Nausea	5 (5.4)	3 (2.5)	0.296
	Misc. medical	7 (7.6)	5(4.1)	0.370
	Misc. surgical	2 (2.2)	1 (0.8)	0.579
	Other	4 (4.3)	6 (5.0)	0.729
	Any	50 (54.3)	57(47.1)	0.334
	Minor complications (Clavien 1-2)	45 (48.9)	45 (37.2)	0.094
	Major complications (Clavien 3-5)	19(20.7)	22(18.2)	0.726

In addition, there was no significant difference in the incidence of post-operative complications (≤90 days post-surgery) between ORC and RRC surgical groups (Table-4). We also stratified complications using the Clavien grading system into major (Clavien 3-5) and minor (Clavien 1-2) subgroups. There was no significant difference in the incidence of either major or minor complications in patients undergoing ORC or RRC procedures (p=0.726 and 0.094, respectively; Table-4).

## DISCUSSION

Surgical intervention with radical cystectomy and extended pelvic lymph node dissection (ELND) is the curative treatment of choice in patients with either muscle-invasive or high-risk non-muscle invasive bladder (2) [Steven & Poulsen; Poulsen et al.; Bi et al. 2015]. In patients with invasive (T2) bladder cancer, ELND has been associated with improved survival compared to standard lymphadenectomy (7, 8). Radical cystectomy with ELND of >16 lymph nodes resulted in a 22% increase in 5-year survival compared to patients in which <16 nodes were excised. In addition, a recent meta-analyses indicated that ELND was associated with a positive effect on recurrence-free survival compared to non-ELND patients (9, 10).

The potential for robotic-assisted surgery to drive a reduction in complication rates, morbidity and mortality associated with cystectomy surgery has received significant interest. As such, whether the use of robotic-assisted surgery compromises the pathological and oncological outcomes relative to open surgery is a key question. Furthermore, it is also important to determine whether indices of perioperative outcome, including blood loss, operative time, and length of stay, are comparable to ORC, since these factors may adversely impact morbidity and recovery time. Finally, complication rates associated with RRC, and how they compare with open surgery, is of clinical relevance. As such, we retrospectively compared these clinical indices in 213 patients who underwent either ORC or RRC over a 10-year contemporary period at our large, urban clinical practice.

Data from a prospective, randomized trial compared oncologic outcomes following open or minimally invasive cystectomy (11). While there was no indication of significant differences in oncologic outcome between surgical modalities, RRC was associated with a reduction in blood loss and shorter hospital stay (11). Looking ahead, the multicenter RAZOR (randomized open versus robotic cystectomy) trial in 320 patients (T1-T4, N0-N1, M0) will be completed in 2016-2017 (12), and will provide additional important data regarding the impact of pelvic lymph node dissection and urinary diversion on oncologic outcomes, complications and health-related quality of life over a 2-year period.

Khan et al. recently described the functional and oncologic outcomes of a small cohort of RRC patients with invasive and non-invasive bladder cancer (14 patients) over a 5-8 year follow-up period. RRC was associated with excellent local disease control, and outcomes in patients with metastatic disease were equivalent to ORC (13). A previous prospective, randomized single center study compared perioperative and pathologic data (EBL, operative time, complications, bowel function and length of stay) in 41 patients undergoing either ORC or RRC (14). The primary aim of this trial was to assess lymph node yield between open and robotic cystectomy. The node yield in both arms was lower than expected for ELND including the common iliac nodes. While this study was not adequately powered to assess secondary endpoints, surgical time was prolonged in the robotic surgery group and surgical blood loss was significantly reduced. While the study cohort was relatively small, these data illustrated that RRC was not inferior to ORC.

The greater lymph node removal in RRC patients may be related to a growing use of extended lymph node dissection and robotic surgery for bladder cancer at our clinical center over the last 3 years. Over the study period, lymph node yield during both open and robotic cystectomy procedures increased at a similar rate. These data support the general trend towards more extensive lymph node dissection that is beneficial in limiting subsequent disease recurrence. The ability to undertake more extensive lymph node removal is likely to have been facilitated by the routine use of robotic-assisted surgical systems. Furthermore, higher lymph node yields (in addition to the potential for selection bias) may be related the lower rate of recurrence in RRC patients in our study.

More recently, Tang et al. described a systematic review of the safety and efficacy of RRC compared to ORC in the treatment of bladder cancer (15). In a meta-analysis of 13 studies (one randomized controlled trial, seven prospective and five retrospective studies) ORC was found to be associated with shorter surgical times. However, patients undergoing RRC experienced significantly fewer complications, less surgical blood loss, shorter length of hospital stay, lower blood transfusion rate, less transfusion needs, shorter time to regular diet, increased lymph node yield and fewer positive lymph nodes. There was no difference in positive surgical margins between ORC and RRC surgical groups. Overall, this meta-analysis demonstrated significant advantages of RRC compared to open surgery. Wang et al. also described the perioperative and pathological outcomes in a small prospective study of 54 patients undergoing either ORC or RRC (16). The 33 patients who underwent RRC had decreased blood loss and requirement for transfusion, but increased surgical duration. The complication rates were similar between groups.

Haber et al. have also published a critical review of laparoscopic and robotic-assisted radical cystectomy surgery between 1992-2007, concluding that RRC combines the patient-recovery advantages of minimally-invasive surgery with the safety of open surgery (17). In general, laparoscopic surgery was associated with reduced blood loss, comparable rates of complications and equivalent oncological outcomes. More recently, a prospective, randomized trial comparing perioperative complications between a total of 124 patients who underwent RRC or ORC failed to identify a large advantage of robotic over open cystectomy (3). Both surgical groups had similar 90-day complication rates, length of stay, pathologic and quality of life outcomes (3).

Importantly, our data illustrate that rates of individual complications in patients undergoing RRC are equivalent to those undergoing ORC. Furthermore, the distributions of major (Clavien 3-5) and minor (Clavien 1-2) complications were not statistically different between surgical groups.

Limitations of our study include its retrospective nature and the potential for selection bias of a patient for a particular surgical procedure. There were no absolute selection criteria for open versus robotic cystectomy. In the early part of our study period (2003-2008), relatively more cases were performed using an open procedure. However, over the study period this changed with robotic cystectomies relatively more common during the last 5 years. As such, there was likely some selection bias, particularly towards open cystectomies in the initial phase of the study period. Positive aspects of our study are that the patient cohort was sampled over a 10-year contemporary time period, and patient has sufficient length of post-surgical follow-up (median 1.4; IQR: 0.6-2.6 years) to allow us to statistically compare recurrence and mortality between surgical groups.

## CONCLUSIONS

While our study was retrospective in nature, and the patient cohorts were relatively small, our data suggest that ORC and RRC patients have comparable post-surgical outcome profiles. RRC may offer additional benefits in terms of lower surgical blood loss and reducing the need for transfusion.
